# Extrapolating microdomain Ca^2+^ dynamics using BK channels as a Ca^2+^ sensor

**DOI:** 10.1038/srep17343

**Published:** 2016-01-18

**Authors:** Panpan Hou, Feng Xiao, Haowen Liu, Ming Yuchi, Guohui Zhang, Ying Wu, Wei Wang, Wenping Zeng, Mingyue Ding, Jianming Cui, Zhengxing Wu, Lu-Yang Wang, Jiuping Ding

**Affiliations:** 1 Key Laboratory of Molecular Biophysics, Huazhong University of Science and Technology, Ministry of Education, College of Life Science and Technology, Wuhan, Hubei, China; 2 Key Laboratory of Image Processing and Intelligent Control, Huazhong University of Science and Technology, Ministry of Education, Department of Biomedical Engineering, College of Life Science and Technology, Wuhan, Hubei, China; 3 Program in Neurosciences and Mental Health, SickKids Research Institute & Department of Physiology, University of Toronto, Toronto, Canada M5G 1X8; 4 Department of Biomedical Engineering, Center for the Investigation of Membrane Excitability Disorders, Cardiac Bioelectricity and Arrhythmia Center, Washington University, St Louis, MO 63130, USA; 5 Department of pharmacology, Soochow University college of pharmaceutical Sciences, Suzhou, 215123, China

## Abstract

Ca^2+^ ions play crucial roles in mediating physiological and pathophysiological processes, yet Ca^2+^ dynamics local to the Ca^2+^ source, either from influx via calcium permeable ion channels on plasmic membrane or release from internal Ca^2+^ stores, is difficult to delineate. Large-conductance calcium-activated K^+^ (BK-type) channels, abundantly distribute in excitable cells and often localize to the proximity of voltage-gated Ca^2+^ channels (VGCCs), spatially enabling the coupling of the intracellular Ca^2+^ signal to the channel gating to regulate membrane excitability and spike firing patterns. Here we utilized the sensitivity and dynamic range of BK to explore non-uniform Ca^2+^ local transients in the microdomain of VGCCs. Accordingly, we applied flash photolysis of caged Ca^2+^ to activate BK channels and determine their intrinsic sensitivity to Ca^2+^. We found that uncaging Ca^2+^ activated biphasic BK currents with fast and slow components (time constants being τ_f_ ≈ 0.2 ms and τ_s_ ≈ 10 ms), which can be accounted for by biphasic Ca^2+^ transients following light photolysis. We estimated the Ca^2+^-binding rate constant k_b_ (≈1.8 × 10^8^ M^−1^s^−1^) for mSlo1 and further developed a model in which BK channels act as a calcium sensor capable of quantitatively predicting local microdomain Ca^2+^ transients in the vicinity of VGCCs during action potentials.

Ca^2+^ ions are perhaps the most important second messenger for triggering a variety of biological functions and often signal in a highly compartmentalized manner. For example, fast release of synaptic vesicles from presynaptic terminals is triggered by Ca^2+^ influx via voltage-gated Ca^2+^ channels during action potentials, and the local high concentration of Ca^2+^ transients at the active zones is critical for gating synchronized fusion of synaptic vesicles (SV) and transmitter release. Experimental measurements of global Ca^2+^ concentrations with Ca^2+^ indicators and mathematical modeling extrapolate that the peak Ca^2+^ concentration seen by the Ca^2+^ sensor on SVs briefly reach tens and even hundreds of micromole levels. Although tremendous progress in recent years has been made with developing novel fluorescent chemical or protein Ca^2+^ sensors, limited spatiotemporal resolution of these sensors presents a major challenge to directly read out local Ca^2+^ transients in real-time.

Large-conductance Ca-activated potassium channels (BK channels), uniquely sensitive to both membrane potential and intracellular Ca^2+^, abundantly distributed in the excitable cells, regulate the membrane excitability and electrical signals in response to the Ca^2+^-influx from the Ca^2+^-permeable channels^1^,^2^. The BK channel encoded by Slo1 gene contains two calcium binding sites in the regulator of conductance for K^+^ (RCK) domains of the caboxy-terminal region^3^,^4^ and may potentially serve as an ideal sensor of local Ca^2+^ rise. However, the affinity of these binding sites is primarily determined under the circumstance of Ca^2+^ uniformly sojourning to its binding sites at equilibrium with very little consideration of dynamics of Ca^2+^ influx or release. Although elaborate Markov models containing multiple parallel open and closed states have been developed to simulate both voltage- and Ca^2+^ dependent gating kinetics of BK channels well^5^,^6^,^7^, the forward binding rate constant of Ca^2+^ (k_b_) remains unknown, making model parameters too unconstrained to meaningfully profile local Ca^2+^ dynamics.

Previous experiments in inside-out patch configuration have attempted to directly measure k_b_ by ultrafast Ca^2+^ concentration jumps via a piezoelectric stepper of two barrel theta pipette^8^,^9^, which enables a solution exchange in less than 1 ms. However, the patch membrane usually invaginates into the pipette tip and forms Ω-shape geometry, slowing the diffusion of Ca^2+^ (~10 ms) to reach the inner face of the membrane patch where the RCK domain of BK channels situates^9^.

To extrapolate the local Ca^2+^ dynamics using BK channels as a sensor, it is therefore necessary to develop a superfast approach of Ca^2+^ delivery mimicking calcium influxes via calcium channels induced by action potentials, and precisely measure k_b_ in order to quantitatively describe the kinetics of BK channels to such fast Ca^2+^ transients. In this study, we have applied laser flash photolysis technique of the caged-Ca^2+^ compound (e.g. NP-EGTA) to achieve instantaneous Ca^2+^ rises, which has been widely used for studying Ca^2+^-dependent processes such as the secretory responses^10^. After a UV flash-induced photolysis, the intracellular calcium concentration have two phases of rise, a fast transient Ca^2+^ rise with peak concentrations up to tens of micromole from the basal [Ca^2+^]_i_ of ~10–200 nM in sub-milliseconds and a slow uniformly steady-state elevation of global [Ca^2+^]_i_ 11,12,13. We took advantage of biphasic properties with laser photolysis of the caged-Ca^2+^ compound to examine both voltage- and calcium-dependent gating behavior, and determined the Ca^2+^ forward binding rate k_b_ for BK. Our results demonstrate that BK channels have higher calcium-sensitivity capable to follow up to tens of μM transient Ca^2+^ changes 0.1–0.2 ms, and established a quantitative model for its utility as the fast local Ca^2+^ sensor to profile the local Ca^2+^ transients during action potential firing.

## Results

### BK-type currents elicited by flash photolysis of caged-calcium showed biphasic activation

**Figure 1:**
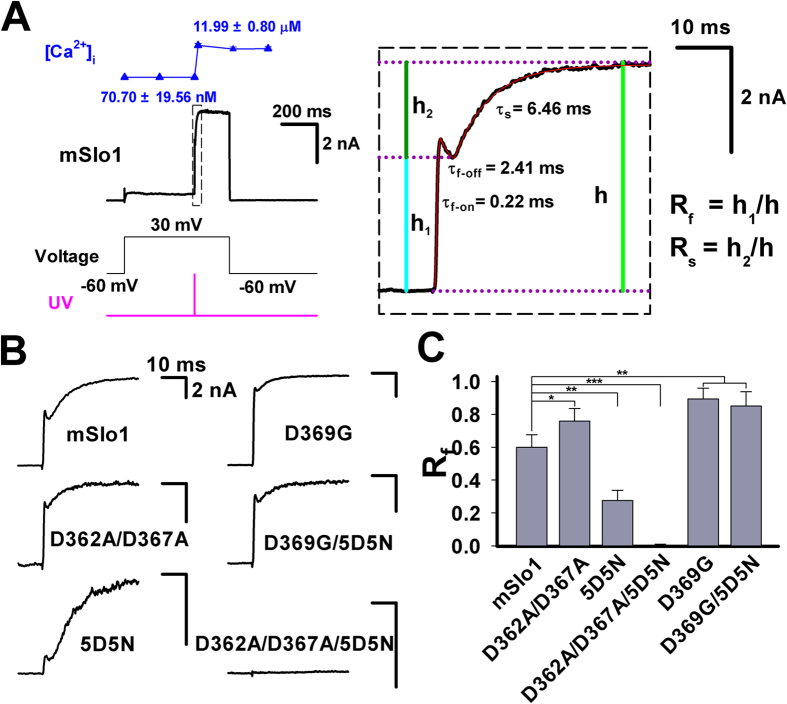
The biphasic currents of BK-type channels elicited by flash photolysis of caged-calcium. (**A**) Trace shows the current from a whole-cell patch from a *HEK293* cell transfected with cDNA encoding mouse *Slo1* α-subunits. The current was stimulated firstly by a voltage step from a 100-ms holding potential of −60 mV to the 600-ms testing voltage of +30 mV, and then by a 0.2-ms UV pulse (pink line) at 500 ms as indicated at the bottom. The measured [Ca^2+^]_i_ in blue triangle is placed at the top. The boxed current elicited by flash showed a biphasic activation with a fast on-time constant τ_f_ = ~0.2 ms, a fast off-time constant τ_f-off_ = ~2.4 ms and a slow time constant τ_s_ = ~10 ms. The proportion of fast and slow components is respectively defined as R_f_ = h_1_/h and R_s_ = h_2_/h as indicated. (**B**) The representative currents, evoked by flash, of mSlo1 and its five mutants as indicated. (**C**) The fast proportion R_f_ of the above currents as shown in (**B**). The R_f_ is 60 ± 7.56% (n = 10) for mSlo1, 75.84 ± 7.68% (n = 10) for D362A/D367A, 25.96 ± 6.20% (n = 10) for 5D5N, 0.0% (n = 10) for D362A/D367A/5D5N and 89.35 ± 6.57% (n = 10) for D369G. Statistical significance for all data was determined using One Way ANOVA (*P < 0.05, **P < 0.01, ***P < 0.001).

To directly investigate the Ca^2+^-sensitivity of BK-type channels, laser UV flashes were used to release Ca^2+^ from caged compound NP-EGTA (10 mM) and activate the currents of mSlo1 and several mutants (BK-type) at various voltages. Fig. 1A shows macroscopic currents of mSlo1 channels expressed in HEK293 cells, which were evoked by a UV flash. The UV flashes with duration-time of 0.2 ms were delivered after the whole-cell configuration was formed for three minutes to ensure the pipette solution uniformly diffused into the cell. After the mSlo1 current reached steady-state at a given voltage of +30 mV, a 0.2 ms UV pulse (pink line) was excited to photolyze the caged Ca^2+^ to increase the intracellular Ca^2+^ concentration([Ca^2+^]_i_) to ~10 μM in less than 1 ms (blue line). The mSlo1 current was further enlarged by the uncaged Ca^2+^ and exhibited a biphasic activation process, presumably as a result of uncaging that produced an early instantaneous transient followed by a plateau increase of intracellular Ca^2+^ concentrations in a spatially uniform manner^13^. Such a complex response appears to be independent of Ca^2+^-release from internal store because preloading cells with thapsigargin (1 μM TG) in recording pipettes for at least 5 min to deplete internal Ca^2+^ stores before flash photolysis has no effect (sFig. 1A). Strikingly, a hooked current appeared at the end of the fast phase. The boxed trace (right) showed typical biphasic activation with the fast on-time (τ_f_ or τ_f-on_), off-time (τ_f-off_) and slow time (τ_s_) constants of ∼0.2, 2.4 and 10 ms, respectively. As an approximation of relative distribution of two components at any given flash, we define the fast and slow proportion of the current as R_f_ = h_1_/h and R_s_ = h_2_/h, respectively (Fig. 1A), where h is the total current. For the summation of two components, we have R_f_ + R_s_ = 1.

### The biphasic activations do not come from different calcium binding sites in RCK domains of BK channels

To examine whether different calcium binding site produces different component, we next made a series of constructs including mutant 5D5N (high-affinity calcium binding site deletion) and the mutant D362A/D367A (low-affinity calcium binding site deletion). Even though both of the 5D5N and D362A/D367A show similar biphasic currents, R_f_(5D5N) is much smaller than R_f_(D362A/D367A) (Fig. 1B). The triple mutant D362A/D367A/5D5N showed no current elicited by uncaged-Ca^2+^ after UV flash (Fig. 1B), indicating that BK channels cannot be activated at 30 mV without calcium binding sites. Furthermore, there was no obviously continuous increase in all the BK-type currents at their steady-state stage, suggesting that uncaged Ca^2+^ did not cause a Ca^2+^-dependent recruitment of BK channels from intracellular pool to membrane surface. This was particularly evident for the triple mutant (D362A/D367A/5D5N) in which a lack of the channel responsiveness to uncaged Ca^2+^ should have not precluded its trafficking presumably derived from Ca^2+^ dependent fusion of vesicles in HEK293 cells. Figure 1C summarizes the some results of R_f_ from mSlo1, aforementioned mutants and several others (sFig. 1B,C). The R_f_ < 1 of all the mSlo1 and mutants, albeit there is a big difference among their values of R_f_, indicates that they all respond to uncaged Ca^2+^ in a biphasic manner. This may imply a lack of direct relationship between the Ca^2+^ binding sites and biphasic waveform of BK currents, as each of Ca^2+^ binding sites produces both phases. Additionally, we found the distinct difference in R_f_ between the mutants L312A and G311I in sFig. 1B,C, i.e., R_f_ (L312A) ≫ R_f_ (G311I), possibly due to their extremely different gating properties^14^.

### The fast proportion of the biphasic activation (Rf) is voltage-dependent but Ca^2+^-independent

**Figure 2:**
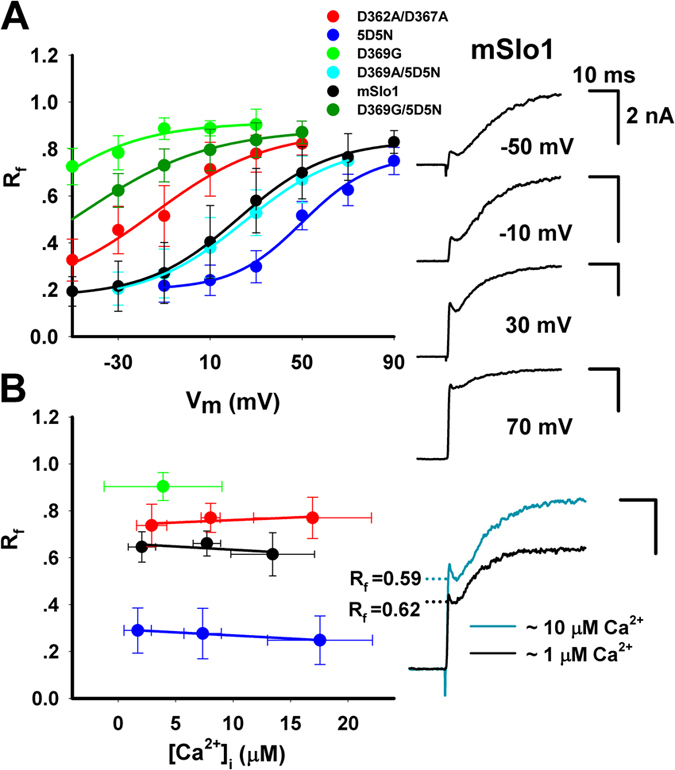
The kinetic characteristics of Rf. (**A**) Voltage-dependence of R_f_ is plotted for mSlo1 and its mutants as indicated. Left, the R_f_-V curves of mSlo1 (black), D362A/D367A (red), 5D5N (blue), D369G (green) and D369A/5D5N (cyan) were fitted to the Boltzmann equation R_f_ = R_fmax_/(1 + exp(V_0.5_−V)/s)), respectively. Here R_fmax_ is the maximal fast proportion, V_0.5_ the voltage of the half maximal fast proportion and s the slope. V_0.5_ and s in mV are 22.5 ± 14.9 and 18.7 ± 5.3 for mSlo1 (n ≥ 8), −10.0 ± 13.0 and 21.2 ± 3.3 for D362A/D367A (n ≥ 6), 50.1 ± 7.7 and 14.9 ± 1.7 for 5D5N (n ≥ 7), −70.6 ± 9.5 and 23.0 ± 2.5 for D369G (n ≥ 5), 24.9 ± 10.3 and 20.5 ± 3.7 for D369A/5D5N (n ≥ 5) and −45.3 ± 8.3 and 23.7 ± 2.8 for D369G/5D5N (n ≥ 5), respectively. Right, the representative currents of mSlo1 were recorded at testing voltage ranging from −50 to 70 mV. (**B**) The Ca^2+^-independence of R_f_ plotted for mSlo1 and its mutants as indicated. Left, [Ca^2+^]_i_ showed little effect on the R_f_ of mSlo1, D362A/D367A and 5D5N at the testing voltage of +30 mV. The data of mSlo1 (black), D362A/D367A (red), D369G (green) and 5D5N (blue) were averaged over three [Ca^2+^]_i_ ranges: 0–5 μM, 5–10 μM and ≥10 μM, and fitted to a straight line. Each slope of mSlo1, D362A/D367A and 5D5N is −0.003 ms/μM, 0.002 ms/μM and −0.003 ms/μM, respectively. Right, the representative currents of mSlo1 were obtained at +30 mV, in the presence of ~1 μM (black) and ~10 μM (dark cyan), respectively.

To determine whether the R_f_ is voltage dependent, we stimulated the currents of mSlo1, 5D5N, D362A/D367A, D369G and D369A/5D5N by flash at various voltage, measured their values of R_f_ and then plotted their R_f_-V curves (Fig. 2A). The shapes of the R_f_-V curves are similar to their G-V curves. After fitted R_f_-V curves to Boltzmann equation, we derived their values of V_0.5_ and slope factor (in mV)[mSlo1: 22.5 and 18.7; 5D5N: 50.1 and 14.9; D362A/D367A: −10.0 and 21.3; D369G: −70.6 and 23.0; D369G/5D5N: −45.3 and 23.5; D369A/5D5N: 24.9 and 20.5] and found that their values of V_0.5_ and slope factors increase by degrees according to the following rank oder: D369G, D369G/5D5N, D362A/D367A, mSlo1, D369A/5D5N, 5D5N, basically in parallel to their corresponding G-V curves, implying that the R_f_ values can mirror differences in the calcium sensitivity (or Ca^2+^ forward binding rate) of BK-type channels and their mutants in response to instantaneous Ca^2+^ rise.

In contrast to the strong voltage-dependence, the Ca^2+^-dependence of R_f_ is not apparent, based on the measured [Ca^2+^]_i_ during flash experiments (Fig. 2B). Moreover, both the fast and slow activation time constants τ_f_ and τ_s_ of BK currents are neither voltage-dependent nor Ca^2+^-dependent (sFig. 2A,B). All the data suggest that the uncaged Ca^2+^ transients in HEK cells under our experimental conditions contain both fast and slow components, consistent with those described in artificial conditions *in vitro*^11^,^12^, although reliable measurement of [Ca^2+^]_i_ can only be made during slow plateau phase (Fig. 1A).

### Determination of a physical calcium binding rate kb

**Figure 3:**
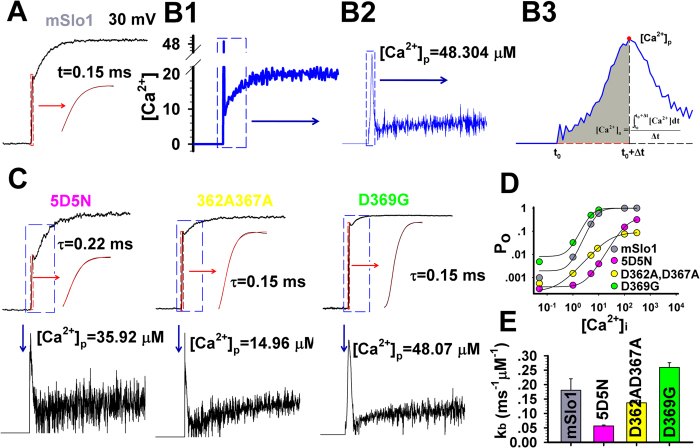
Estimation of the Ca^2+^ binding rate constants of BK-type channels. (**A**) Trace of mSlo1 currents was evoked by a UV flash in the whole-cell mode at 30 mV. The red box shows the fast rising current of mSlo1 with a time constant τ = 0.15 ms by a fit to eq. (3). (**B1**) The Ca^2+^-releasing time course by the UV flash was calculated from the 10-state mSlo1 model. The algorithm is described in sFig. 3. (**B2**) The details of blue box in (**B1**). Here [Ca^2+^]_p_ denotes the peak value of [Ca^2+^]_i_. (**B3**) The averaged rising calcium concentration in the blue box of (B2) is calculated by a formula as shown in inset. (**C**) Top, the current traces of mSlo1 were evoked by uncaged Ca^2+^ at −30, 10 and 50 mV as indicated. The details in red box are shown in inset as indicated by arrows. The values of τ were derived from a fit to eq. (3). Trace is black and fit red. Bottom show the detailed Ca^2+^-releasing time course in the top blue boxes, indicated by blue arrows. (**D**) The P_o_-[Ca^2+^]_i_ dose-response curves of BK channels at 30 mV were plotted for mSlo1, 5D5N, D362AD367A and D369G, respectively. Their dissociattion equilibrium constant K_d_ values were listed in sTable2. (**E**) Based on Eq. 4, k_b_ = 0.18 ± 0.04 (n = 6) for mSlo1, 0.057 ± 0.003 (n = 5) for 5D5N, 0.014 ± 0.01 (n = 4) for D362AD367A and 0.26 ± 0.02 (n = 5) for D369G, respectively, at 30 mV (sTable 2).

In previous models for BK-type channels in10-state gating scheme^15^ (sFig. 3, sTable 1), the apparent Ca^2+^ binding rate k_s_ was arbitrarily set as 1 in ms^−1^ μM^−1^ under an empirical assumption of Ca^2+^ forward binding rate being around 10^9^ * s^−1^M^−1^ for many Ca^2+^ binding proteins including BK channels. Hence, all rate constants that allow optimal fits of experimental data remain physiologically irrelevant, unless Ca^2+^ forward binding rate (kb) is fixed to constrain relative changes in the open (k_o_) and closed (k_c_) dissociation equilibrium constants, which hold the k_o_/k_c_ constant. To acquire the physical Ca^2+^ binding rate k_b_, we only consider a simplest two-state 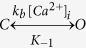 model of ligand (Ca^2+^) and receptor (BK) to describe the BK currents evoked by the uncaged Ca^2+^ release, which can be seen as an unit model of the MWC model of BK. Here the C is a closed state, and the O an open state. The forward rate between C and O is Ca^2+^-dependent. Based on the C-O model, we have the calcium binding rate constant k_b_ = 1/(τ * ([Ca^2+^]_a_+k_d_)) in μM^−1^ ms^−1^ (eq. (4)), where k_d_ is the dissociation equilibrium constant, [Ca^2+^]_a_ the mean Ca^2+^ concentration and τ the activation time constant during the fast rising phase. Given all the τ, [Ca^2+^]_a_ and k_d_, we can calculate the calcium binding rate constant k_b_ of BK-type channels. As a calculated Ca^2+^ spike was always appearing at the initial phase of Ca^2+^ uncaging (sFigs 3 and 8 and [Ca^2+^]_i_ calculation in Online Methods), we define the [Ca^2+^]_a_ as an averaged [Ca^2+^]_i_ over the fast rising time of uncaged Ca^2+^ pulse (Fig. 3A–B3). During the rising period of uncaged Ca^2+^, we calculated the fast rising time constant τ of BK currents, based on a C-O model (eq. (3) in Online Methods). For instance, we obtained the τ = 0.15 ms at 30 mV by a fit of mSlo1 current to eq. (3) (Fig. 3A), the [Ca^2+^]_a_ = 20.89 μM, averaged from 0 to a Ca^2+^ peak value [Ca^2+^]_p_ = 48.30 μM (Fig. 3B1–B3). By the same logic, we estimate τ to be ~0.15–0.30 ms with a [Ca^2+^]_a_ = ~10–20 μM for other BK-type currents (Fig. 3C). The values of k_d_ for mSlo1 and its mutants can be readily obtained from Ca^2+^ dose-response curves of BK-type channels (Fig. 3D, sTable 2). Based on the Eq. (4), we thus derived the values of k_b_ at 30 mV as 0.18 ± 0.04 (n = 6) for mSlo1, 0.057 ± 0.003 (n = 5) for 5D5N, 0.014 ± 0.01 (n = 4) for D362AD367A and 0.26 ± 0.02 (n = 5) for D369G, respectively, as indicated in sTable 2 and Fig. 3E. Here, the k_b_ value of mSlo1 is near to the typical limitation of calcium binding rates 10^8^ * s^−1^M^−1^ 16. With this new k_b_, we now for the first time able to constrain the BK model with physiologically meaningful parameters (sFig. 7, sTable 3) and yield optimal match between all the fits and the current traces of mSlo1 channels to drive subsequent non-stationary calculation of local Ca^2+^ transients.

### The BK channels can be used as a Ca^2+^ sensor to extrapolate the local intracellular Ca^2+^ from VGCCs in the vicinity of BK channels during action potentials

**Figure 4:**
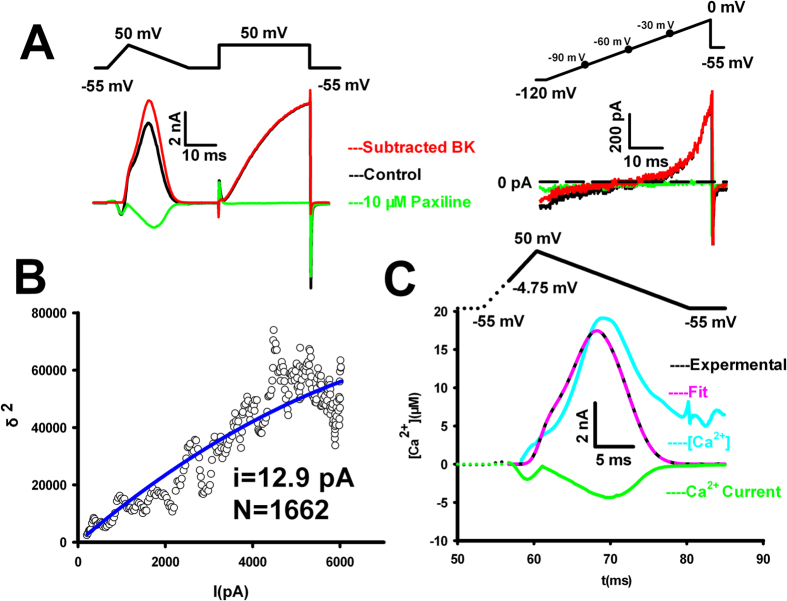
Calculating the local intracellular Ca^2+^ based on BK currents. (**A**) Left top, the voltage protocol, composed of the waveforms of a pseudo-action-potential (pseudo-AP) and a voltage step to 50 mV, was designed to record the Ca^2+^-activated currents of BK channels as to acquire the channel number, co-expressed with Cav1.2 in *HEK293* cells. Left bottom, the current (black) were obtained in whole-cell patch bathed in the normal saline from a *HEK293* cell co-expressed mSlo1 with Ca_v_1.2 subunits. The current (green) was recorded after applying10 μM Paxilline, of which major component is Ca^2+^ current. The pure BK current (red) was subtracted from the blocked current (green). Right top, a ramp voltage protocol was designed to determine the reversal potential of BK channels in the same case. Right bottom, the current was obtained with no Paxilline (black), with 10 μM Paxilline (green) and the pure BK current was obtained from their difference (red). The holding potential is set at −60 mV to best mimic the resting potential (~−60 mV co-expressing both channels) and minimize the leak or holding currents for voltage-clamp experiments so as to ease the current subtraction procedure for amplitude measurements. (**B**) Analysis of BK current variance. The scatter plot was obtained from the current variance versus the mean BK currents recorded by a voltage step to 50 mV in (**A**). Fitted curve (blue) represents the equation: σ^2^ = iI − I^2^/N with the parameters i = 12.9 pA at 50 mV and N = 1662, where σ^2^ represents the variance of BK currents, i the single-channel current amplitude, I the mean current and N the number of BK channels. (**C**) Estimation of local intracellular [Ca^2+^]_i_. Fitting the 10-state BK model shown in sFig 3 to the pure BK current evoked by the pseudo-AP to determine the intracellular [Ca^2+^]_I_ (cyan). The black point denotes experimental values of BK currents and the pink line is a fit. Green represents the Ca^2+^ current. Voltage protocol used for calculation was plotted at the top, which is composed of dotted and solid lines, indicating that the initial point for calculation started at −4.75 mV to avoid the noise signal as indicated.

The k_b_ value of mSlo1 is over 10^8^ * s^−1^M^−1^, indicating that the BK channel is highly sensitive calcium sensor compared to those mediating the fusion vesicles in chromaffin cells or SVs in central synapses^12^,^17^. Such a fast binding rate for Ca^2+^ to activate the BK channel justifies it as an optimal Ca^2+^ sensor to extrapolate the local intracellular Ca^2+^ from VGCCs in the vicinity of BK channels during action potentials (APs). As described previously, using the kinetic model for fitting BK current activated by uncaged Ca^2+^, we can reversely extrapolate the local Ca^2+^ profile. To obtain a proof of principle, we transfected Cav1.2 channel alone or in combination with mSlo1 and confirmed that both channels were successfully expressed in HEK293 cells (sFig.9). In cells co-expressed mSlo1 and Cav1.2, we estimated the number of BK channels and Ca^2+^ channels using a voltage protocol consisted of a pseudo-AP and voltage-step to determine the single-channel current and the number in cells by the mean-variance analyses of BK currents (Fig. 4A, left) as well as their reversal potential under physiological condition (Fig. 4A, right). To extract the pure BK current as it may be mixed with Ca^2+^ currents, we applied Paxilline, a specific blocker of BK channels to digitally separate distinct ionic components. Based on analysis of BK current variance, the single-channel current (i) was estimated to be 12.9 pA and the channel number N was 1662 in each cell (Fig. 4B). In Fig. 4C, fitting to BK current evoked by pseudo-AP, we obtained the local intracellular Ca^2+^ dynamics which closely follows Ca^2+^ currents and reaches a peak concentration of >20 μM Ca^2+^and rapidly declines to ∼4 μM Ca^2+^ upon repolarization of the pseudo-AP. These observations suggest that BK channels were located within the vicinity of calcium channels and faithful track the rise and fall of local Ca^2+^ transients.

These observations also provide important insights into the coupling relationship between VGCCs and BK channels or their distance. Because I _Cav1.2_ (0 mV) = −1529 pA with a single-channel conductance of 2.4 pS (at a physiological external concentration of 2 mM [Ca^2+^]_o_)^18^ and a driven force of 60 mV with 100% open probability of CavL channels, we can approximate N(Cav1.2) = 10618 per cell. Given the mean total capacitance of a HEK293 cell about 12 pF and the membrane capacitance C_m_ = 1 μF/cm^2^ 16, the cell surface area is 1200 μm^2^. In other words, the Cav1.2 density is 8.8 Cav1.2/μm^2^. As N(BK) = 1662 (Fig. 4B), the BK density is 1.4 BK/μm^2^. Assuming random distribution of both channels in HEK cells, we can proximate that each BK channel is surrounded by 6–8 Cav1.2. Considering a total of 24 μM Ca^2+^ during the single AP firing, we can extrapolate that each BK channel is supported with 3–4 μM Ca^2+^ per VGCC. In the case of the intracellular 5 mM EGTA, their coupling distance must be within the microdomain of about 60–80 nm, on the basis of established Ca^2+^ buffer models that quantitatively describe the relationship for Ca^2+^(μM)-r(distance)^19^.

## Discussion

The BK channel, abundant expressed in a wide range of cells and tissues, is the only channel rapidly responding to the membrane changes induced by both the voltage and Ca^2+^ in a tremendously wider range (V≤ ±200 mV or unlimited and nM-mM Ca^2+^). In this study, we established the frame work for these channels to act as an optimal Ca^2+^-sensor to track fast Ca^2+^ transients in real time. Although many organic and protein Ca^2+^ sensors have been developed to image Ca^2+^ dynamics in cells with increasing spatiotemporal resolution, it is not only labor intense but also worrisome that the Ca^2+^ homostasis is perturbed with addition of exogenous reagents and Ca^2+^ binding proteins. In contrast, BK channels are native to many cells particularly neurons and synapses, we suggest these channels have a broad utility for detecting and profile Ca^2+^ transients important for local and compartmentalized signaling.

Unlike other studies on steady-state kinetics of BK, we studied the dynamics of BK in response to non-stationary Ca^2+^ transients. By means of laser flash photolysis techniques in this study, we delineated properties of BK currents such as the biphasic activation, voltage-dependent R_f_(V) as well as the voltage- and calcium- independent time constants τ_f_ and τ_s_ in response to Ca^2+^ transients that closely resemble physiological Ca^2+^ influx through voltage-gated calcium channels or release from internal stores. We determined the calcium forward binding rate k_b_ for BK channels and advanced previously established 10-state BK model with physiologically relevant rate constants. To our best knowledge, the kb value (10^8^ * s^−1^M^−1^) for BK channels is among the fastest forward binding rates, comparable to fast Ca^2+^ buffer BAPTA and calmodulin, but 1 or 2 orders of magnitude faster than slow Ca^2+^ buffer EGTA and Ca^2+^ binding proteins calbindin and calretinin^20^,^21^,^22^,^23^. Because fast Ca^2+^ bindings are directly coupled to BK channel openings, currents can readily serve as a sensitive readouts of local Ca^2+^ transients, presenting advantages over calmodulin based protein Ca^2+^ sensors (i.e. GCAMPs)^24^. Indeed, using the BK current activated by the Ca^2+^ influx of Cav1.2 channels co-expressed in HEK cells, we calculated the time course of local Ca^2+^ in millisecond resolution during a pseudo-AP and the distance between Cav1.2 and BK. These results provide a solid foundation for further exploration of local Ca^2+^ transients and downstream coupling targets in native cells.

Similar to the experiments of UV-flash uncaging Ca^2+^, the k_b_ of mSlo1, D369G and 5D5N except for D362A/D367A are consistent to the rank order of R_f_, indicating that the greater the R_f_, the greater the binding rate k_b_. The anomalous behavior of R_f_(D362A/D367A) may come from the specific gating of mSlo1(D362A/D367A), but further studies are needed to discern its unique properties. Different rates of Ca^2+^-dependent activation of these mutants may also expand their utility to profile local Ca^2+^ dynamics in cases where native tissues do not express BK or low level of voltage-gated Ca^2+^ channels by cell-specific gene targeting approaches with the aim of minimal perturbation to physiologically functions.

Using the genetic method, we demonstrated that the biphasic currents of BK induced by UV flash is not due to different affinity of Ca^2+^ binding sites of BK-RCK domains, and instead likely originate from biphasic calcium transients as previously demonstrated^11^,^12^. Dynamic interactions between the robust endogenous calcium buffers and uncaged Ca^2+^ rise inside cells may underlie such a process^11^. We also noticed that large fast Ca^2+^ transients induced relatively small BK hook currents, suggesting that the Ca^2+^ binding rate k_b_ to BK is the rate-limiting parameter for follow rapid time course of Ca^2+^ transients during brief APs. It can be envisaged that new mSlo1 mutants with faster kb can be developed in future studies to overcome the rate limit and more closely track Ca^2+^ dynamics in real-time.

Taken together we have developed a novel method to calculate the time course of local Ca^2+^ by measuring BK currents. The core of this approach is to accurately calculate the number of BK channels and to acquire the pure currents of BK using the specific inhibitors of BK. In most cases, the channel number can be obtained by either variance analysis or steady-state current at a known Ca^2+^ concentration. In cases where the number and topographic distribution of Ca^2+^ channels have been mapped out, BK may not only serve as a sensitive sensor of local Ca^2+^ transients but also coupling distance of their micro-/nano-domains in native cells such as neurons and synapses. The utility of BK channels as Ca^2+^ sensor in native cells depends critically on the distance between BK channels and Ca^2+^ channels/stores. The distance measurement can be experimentally determined by using electron microscopy of immune gold particles of different size labeling BK channels and calcium channels in the same preparation. Recent developments in super-resolution microscopy may potentially help determine such distances directly if transgenic knock-in mice with BK and calcium channels tagged with different fluorescent proteins are created. Alternatively, the distance can be quantitatively calculated by injecting different concentrations of Ca^2+^ buffers such as EGTA and BAPTA into cells to test the degree of attenuation of BK currents. Although these two buffers have similar equilibrium dissociation constants (Kd), the forward binding constant of EGTA is nearly 100 times slower than BAPTA, and hence not effective in capturing Ca^2+^ ions from sources that are within nanodomain distance (<100 nm) whereas BAPTA will be. On the contrary, if Ca^2+^ source are relatively far in microdomain distance (>100 nm), both EGTA and BAPTA can work equally well. Using linearized buffered Ca^2+^ diffusion models^19^,^26^, one can estimate the mean distance between BK channels and Ca^2+^ source to enable quantitative profile of brief Ca^2+^ transients in real time during biological activity.

## Online Methods

### Cell culture and Transfection

HEK293 cells were cultured in modified Eagle’s medium (DMEM, Gibco) supplemented with 10% fetal bovine serum (FBS, Gibco) at 37 °C incubator with 5% CO_2_. The day before transfection, cells were transfered into a 24-well plate and transiently transfected using lipofectamine 2000 (Invitrogen) according to manufacturer’s protocol. Recordings were carried out in 1–2 days after transfection.

### Solutions

All the flash experiments were performed under the normal saline in bath contained the following (in mM): 140 NaCl, 5 KCl, 1.8 CaCl_2_, 2 MgCl_2_, 10 HEPES and 10 Glucose with the PH adjusted to 7.4. Pipette solution contained the following (in mM): 120 KCl, 10 NP-EGTA, 8.05 CaCl_2_, 2 K_2_ATP, 0.4 Fura-4f and 0.4 Mag-Fura-2 and 10 HEPES with the pH adjusted to 7.2. For the experiments of BK current activated by calcium influx, the extracellular solution contained the following (in mM): 150 NaCl, 4 KCl, 2 CaCl2, 1 MgCl2, 10 HEPES (pH 7.38); Pipette solution contained the following (in mM): 140 KCl, 10 NaCl, 5 EGTA, 1 MgCl2, 10 HEPES (pH 7.36). For the experiments of BK kinetic modeling, intracellular solutions with different free Ca^2+^ were made by mixing (in mM) 160 MeSO3K and 10 HEPES with 5 EGTA (for 0 μM Ca^2+^); 5 EGTA and 3.25 CaCl2 (for 1 μM Ca^2+^); 5 HEDTA and 2.99 CaCl2 (for 10 μM Ca^2+^); 0.1 CaCl2 (for 100 μM Ca^2+^); 0.3 CaCl2 (for 300 μM Ca^2+^), with the pH adjusted to 7.0. Free Ca^2+^ was estimated by the EGTAETC program (E. McCleskey, Vollum Institute, Portland, OR). Pipette solution contained the following (in mM): 160 MeSO_3_K, 2 MgCl_2_, 10 HEPES with the pH adjusted to 7.0. All the chemicals were attained from Sigma except that NP-EGTA, Mag-Fura-2 and Fura-4f were from Invitrogen.

### Electrophysiology

Patch pipettes pulled from borosilicate glass capillaries with resistance of 2–4 megohms when filled with pipette solution. Macroscopic currents were recorded 3 minutes after the whole-cell patch formed, and another 3 minutes before the next flash in the same patch. All the flash experiments were performed using the EPC-9 patch-clamp amplifier and corresponding software (HEKA, Germany). Currents were typically digitized at 50 kHz and filtered at 8.9 kHz (Bessel) to reduce the impact caused by filter settings^27^. UV excitation light source(Rapp OptoElectronic, Germany) was used to uncage the intracellular Ca^2+^, and calcium concentration signals were recorded by measuring the fluorescence ratios of 340/380 nm light provided by monochromatic light source(TILL Photonics, Germany). During the Flash experiments recordings, the laboratory should be kept in a dark environment to prevent light pollution, and both of the inside-out and the whole-cell patch experiments were performed in normal saline solutions. All experiments were performed at room temperature (22–24 °C).

### Western Blot

mSlo1 and C-HA tagged Ca_v_1.2 in pcDNA3.1 were co-expressed in HEK293 cells, 24 hrs after transfection, the cells were lysed (lysis buffer contained 20 mM Tris-HCl/pH 7.5, 150 mM NaCl, 1% NP-40, 0.1% Triton X-100, 0.2 mM phenylmethylsulfonyl fluoride and protease inhibitors. After vertical rotated at 4 °C for 1 h, the lysed cells then were high-speed centrifuged (12, 000 rpm) at 4 °C for 30 min. The supernatants were then added loading buffer and boiled at 60 °C for 10 min. Proteins in the lysate were separated on polyacrylamide gels and transferred to a nitrocellulose membrane. After blocking with 5% nonfat milk in 0.1% Tween 20 in Tris-buffered saline, the blots were probed with mouse monoclonal anti-Slo1 antibody (abcam, ab99046) and mouse monoclonal anti-human HA antibody (Millipore, 05-904), respectively. Horseradish peroxidase-coupled goat anti-mouse IgG was used as the secondary antibody for the blots. The membrane was washed with 0.1% Tween 20 in Tris-buffered saline, and proteins were visualized with an enhanced chemiluminescence detection system.

### Data analysis and simulation

Recording data were analyzed with IGOR (Wavemetrics, Lake Oswego, OR), Clampfit (Axon Instruments, Inc.) and Sigmaplot software (SPSS, Inc.). Unless stated otherwise, the data are presented as mean ± S.D. Calculations of parameters for the kinetic modeling were solved numerically, using a Q-matrix and a particle swarm optimization-golden section search (PSO-GSS) algorithms. The global fitting routines were written and executed with software CeL (Huazhong University of Science and Technology, Wuhan, Hubei, China), compiled with the C++ compiler to run under Windows XP.

The G-V curves of BK-type channels were fitted to the single Boltzmann equation:


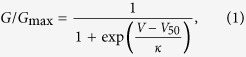


where V_50_ is the voltage at which the conductance (G) is half the maximum conductance (G_max_) and κ is a factor affecting the steepness of the activations. The equilibrium open probability P_o_ can be written as^5^:


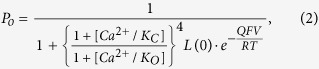


where, [Ca^2+^] is the intracellular calcium concentration, K_C_ the closed Ca^2+^ dissociation constant, K_O_ the open Ca^2+^ dissociation constant, F Faraday’s constant, R the universal gas constant, T temperature, V voltage, Q the equivalent gating charge associated with the closed-to-open conformational change, and L(0) the open-to-closed equilibrium constant in the absence of bound Ca^2+^ at 0 mV.

For the 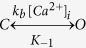 model, we have


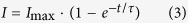



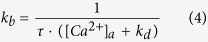


where *I* denotes the BK current, *I*_*max*_ the maximum BK current, a the amplitude of BK current, *τ* the activation time constant during the fast rising phase, *k*_*b*_ the calcium binding rate constant, *k*_*d*_ the dissociated equilibrium constant and [*Ca*^2+^]_*a*_ the averaged intracellular [*Ca*^2+^]_*i*_.

### [Ca^2+^]_i_ calculation

Kinetic modeling is an important method for better understanding the electrophysiological characteristics of ion channels. In kinetic modeling, the membrane current *I*(t) can be depicted as a function of membrane voltage *V*(t) and calcium concentration [*Ca*^2+^]_i_(t),


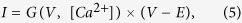


where *G*(*V*, [*Ca*^2+^]_i_) represents the whole-cell conductance determined by both the voltage and calcium concentration and *E* the reversal potential of channels. Unlike the Hodgkin-Huxley model, the total conductance is described as follows:


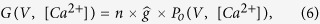


where *n* is the number of ion channels on the membrane, *ĝ* the single-channel conductance, and *P*_*o*_(*V*, [*Ca*^2+^]_i_) the open probability, which can be solved from the kinetic models of channels with numerical method of differential equation (Runge-Kutta or Q-Matrix).

Combining eq. (5) and eq. (6), we can get


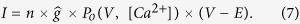


In eq. (7), given *I, n, ĝ, V, E* and *P*_*o*_(), the instantaneous [*Ca*^2+^]_i_ can be determined. However, unlike *I, V, E*, and *ĝ, P*_*o*_(*V*, [*Ca*^2+^]_i_), *n* is hardly to be obtained directly through existing experimental techniques. In this paper, a novel three-step method is proposed to obtain the instantaneous [*Ca*^2+^]_i_ value. Firstly, *P*_*o*_() is determined based on the Monod-Wyman-Changeux (MWC) model. Then, *n* is got through the steady-state calculation of eq. (7). Finally, the instantaneous [*Ca*^2+^] can be obtained through eq. (7). The details of method are executed in three steps as follows (sFig. 8A):

1. Step one: establishing the MWC model for BK-type channels and calculating its open probability *P*_*o*_(*V*, [*Ca*^2+^]_i_).

Models of BK-type channels are an allosteric MWC model of 10-state C5-O5 with 11-parameters, which reflects the voltage- and Ca^2+^-dependent open probability (*P*_*o*_(*V*, [*Ca*^2+^]_i_)). Firstly, a set of activated and deactivated data at different [*Ca*^2+^]_i_ level (fits) were fitted to a MWC model to determine the eleven unknown parameters with the software CeL^15^ (sFig. 8A). This model can be used to calculate the channel *P*_*o*_(*V*, [*Ca*^2+^]_i_).

(2) Step two: determining the channel number *n* in eq. (7)

This step is performed at steady state. From the eq. (7), the steady-state current *I*_*steady*_*(∞)* can be depicted as


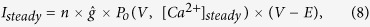


where *I*_*steady*_, [*Ca*^2+^]_i-*steady*_, *V* and *E* were measured from experiment. *P*_*o*_(*V*, [*Ca*^2+^]_i_) was determined from the model in step 1. Therefore, we got *n* from the eq. (8).

3. Step three: Instantaneous [*Ca*^2+^]_i_ calculation

After step 1 and 2, the instantaneous [*Ca*^2+^]_i_ can be determined based on the eq. (7) with all other known parameters. However, the analytical solution of the instantaneous [*Ca*^2+^]_i_ is difficult to obtain because *P*_*o*_(*V*, [*Ca*^2+^]_i_) is in a form of complex differential equation system.

Here, instantaneous [*Ca*^2+^]_i_ calculation is treated as a numeric optimization problem, where [*Ca*^2+^(*t*)]_i_ is the only parameter to be optimizes. Here the Q-Matrix method is used to calculate the values of *P*_*o*_(*t*) and *I*(*t*) at the time *t*, and then to minimize the error between the calculated *I*(*t*) and the actual *I*(*t*) by an optimization algorithm of Evolution Strategy (ES).

The ES is a class of numeric optimization techniques based on the ideas of adaption and evolution. A group of floating point solution candidates evolves with search operators, such as selection, recombination and mutation. In common with simple genetic algorithms, the operators are applied in a loop. An iteration of the loop is called a generation. The sequence of generations is continued until a termination criterion is met. The flowchart of ES is shown in sFig. 8B.

When calculation begins, [*Ca*^2+^(*t*)]_i_ measured at time 0 in experiment is set as the initial steady-state [*Ca*^2+^]_i_. Then [*Ca*^2+^(*t*)]_i_ at next time step can be got through ES optimization. This process goes step by step until [*Ca*^2+^(t)]_i_ at all time step is gotten.

It is obviously that the method in step 2 used to determine the channel number in the UV-flash case completely differs from the variance method^25^ used in the pseudo-AP case.

## Additional Information

**How to cite this article**: Hou, P. *et al.* Extrapolating microdomain Ca^2+^ dynamics using BK channels as a Ca^2+^ sensor. *Sci. Rep.* **6**, 17343; doi: 10.1038/srep17343 (2016).

## Supplementary Material

Supplementary Information
