# Supplementary material for: Extrapolating microdomain Ca2+ dynamics using BK channels as a Ca2+ sensor
Source: Sci Rep. 2016 Jan 18;6:17343. doi: 10.1038/srep17343 (PMC4726033; doi:10.1038/srep17343)
Supplement: Supplementary Information [file srep17343-s1.doc]

**Extrapolating microdomain Ca^2+^ dynamics using BK channels as a Ca^2+^ sensor**

Panpan Hou^1,4,§^, Feng Xiao^2,§^, Haowen Liu^1,§^, Ming Yuchi^2,§^, Guohui Zhang^4^,Ying Wu^1^, Wei Wang^1^, Wenping Zeng^1^, Mingyue Ding^2^, Jianming Cui^4,5^,Zhengxing Wu^1^*, Lu-Yang Wang^3^,* and Jiuping Ding^1^,*

1. Key Laboratory of Molecular Biophysics, Huazhong University of Science and Technology, Ministry of Education, College of Life Science and Technology, Wuhan, Hubei, China.
2. Key Laboratory of Image Processing and Intelligent Control, Huazhong University of Science and Technology, Ministry of Education, Department of Biomedical Engineering, College of Life Science and Technology, Wuhan, Hubei, China.
3. Program in Neurosciences and Mental Health, SickKids Research Institute & Department of Physiology, University of Toronto, Toronto, Canada M5G 1X8
4. Department of Biomedical Engineering, Center for the Investigation of Membrane Excitability Disorders, Cardiac Bioelectricity and Arrhythmia Center, Washington University, St Louis, MO 63130, USA
5. Department of pharmacology, Soochow University college of pharmaceutical Sciences, Suzhou, 215123, China

^§^ These authors contributed equally to this work.

* To whom correspondence should be addressed.

Jiuping Ding<[jpding@mail.hust.edu.cn](mailto:jpding@mail.hust.edu.cn)>

Lu-Yang Wang<luyang.wang@utoronto.ca>

Zhengxing Wu <ibbwuzx@mail.hust.edu.cn>

**Supplemental Material**

**Figure Legends**

**sFig. 1. Intracellular stores showed no effect on the biphasic calcium-gating behavior by flash.** (A) The mSlo1 current elicited by flash was obtained at +30 mV after treated with 1 μM TG for 5 minutes. (B) the L312A current elicited by flash was obtained at +30 mV. (C) The G311I current elicited by flash was obtained at +80 mV.

**sFig. 2. The voltage- and calcium-independence of activation time constants elicited by flash. (**A) Activation time constant τ is plotted against membrane potentials. The τ_f_ and τ_s_ denote the fast time constant and slow time constant, respectively. (B) Activation time constant τ is plotted against the intracellular Ca^2+^. The slopes for mSlo1, D362A/D367A and 5D5N are -0.38 ms/μM, -0.34 ms/μM and -0.38 ms/μM, respectively. The representative traces of mSlo1 channels are placed at the right sides.

**sFig. 3. Determination of the [Ca^2+^]_i_ from the currents of mSlo1 evoked by flash. (**A) The 10-state kinetic model used for BK-type channels, such as, mSlo1, 5D5N, D362A/D367A and D369G channels. All the parameters are listed in sTable 1. (B) The activation and deactivation currents (black) of mSlo1 were recorded in inside-out patches at 0, 1, 10 and 300 μM Ca^2+^, respectively. Red traces are simulations from the 10-state mSlo1 model shown in (A). (C) The G-V curves of mSlo1 are plotted for the data (black) and simulations (red) shown in (B). (D) The biphasic currents (black) of mSlo1 channels were elicited by flashes at different voltages as indicated. The fits (red) of corresponding biphasic currents were calculated, respectively, based on the method as described in sFig. 3A-B. The details in the black box are shown in the insets. (E) The calculated intracellular calcium concentrations produced by flash photolysis are shown under the corresponding currents. The slow rising time constants are fitted by a single exponential function. For methods, please see the Appendix and sFig. 3.

**sFig. 4. Determination of the [Ca^2+^]_i_ from the currents of 5D5N evoked by flash.** Except for 5D5N, all is the same as described in Fig. 3.

**sFig. 5. Determination of the [Ca^2+^]_i_ from the currents of D362A/D367A evoked by flash.** Except for D362A/D367A, all is the same as described in Fig. 3.

**sFig. 6. Determination of the [Ca^2+^]_i_ from the currents of D369G evoked by flash.** Except for D369G, all is the same as described in Fig. 3.

**sFig. 7. Fitting 10-state model to the currents of mSlo1 channels with the physical binding constant k_b_ shown in sTable. 3**. (A1) fitting 10-state model to the activation and deactivation currents of mSlo1 with k_b_ = 1.8*10^8^ M^-1^s^-1^, in the presence of 0, 1, 10 and 300 μM Ca^2+^, as indicated. (A2), the G-V curves were plotted for mSlo1. The fits are red, and the data black. (B1-D1) Except for D369G, D362A367A and 5D5N mutant respectively, all is the same as described in (A1). (B2-D2) Except for D369G, D362A367A and 5D5N mutant respectively, all is the same as described in (A2). The detailed parameters for mSlo1, 5D5N, D362AD367A and D369G are listed in sTable 3.

**sFig. 8. The algorithm of uncaged calcium concentration induced by the UV flash.** (A) The flowchart of proposed method for calculating local [Ca2+]. (B) The flowchart of the optimization algorithm of Evolution Strategy (ES) used in local [Ca2+] calculation.

**sFig. 9. The mSlo1 or Cav1.2 channels were successfully transfected in HEK293 cells.** (A) Top, the same voltage protocol as shown in Fig.4A. Bottom, the current (black) was stimulated by the above voltage protocol in whole-cell patch bathed in the normal saline from a HEK293 cell expressed mSlo1 channel alone. The current was recorded after applying10 μM Paxilline (green) or 1 mM CdCl2 (dark green). (B) The current (black) were stimulated by the same voltage protocol as shown in sFig.9A in whole-cell patch bathed in the normal saline from a HEK293 cell expressed Cav1.2 channel alone. The current (dark green) was obtained after applying 1 mM CdCl2. The scale bars were the same as sFig.9A. (C) The expression of mSlo1 and Cav1.2 in HEK293 cells. mSlo1 and Cav1.2 in pcDNA3.1 were co-expressed in HEK293 cells, 24 hrs after transfection, the cells were lysed (lysis buffer contained 20 mM Tris-HCl/pH 7.5, 150 mM NaCl, 1 % NP-40, 0.1 % Triton X-100, 0.2 mM phenylmethylsulfonyl fluoride and protease inhibitors. After vertical rotated at 4 °C for 1 h, the lysed cells then were high-speed centrifuged (12, 000 rpm) at 4 °C for 30 min. The supernatants were then added loading buffer and boiled at 60 °C for 10 min. Proteins in the lysate were separated on polyacrylamide gels and transferred to a nitrocellulose membrane. After blocking with 5 % nonfat milk in 0.1 % Tween 20 in Tris-buffered saline, the blots were probed with mouse monoclonal anti-Slo1 antibody (Abcam, ab99046) and rabbit polyclonal anti-Cav1.2 antibody (Abcam, ab58552), respectively. Horseradish peroxidase-coupled goat anti-mouse IgG and goat anti-rabbit IgG were used as the secondary antibody for the blots, respectively. The membranes were washed with 0.1 % Tween 20 in Tris-buffered saline, and proteins were visualized with an enhanced chemiluminescence detection system. The lanes labeled “null” were loaded the lysate of untransfected HEK293 cells, and the lanes labeled “mSlo1+Cav1.2” were loaded the lysate of HEK293 cells co-transfected mSlo1 and Cav1.2.

**Figures**

**sFig. 1**


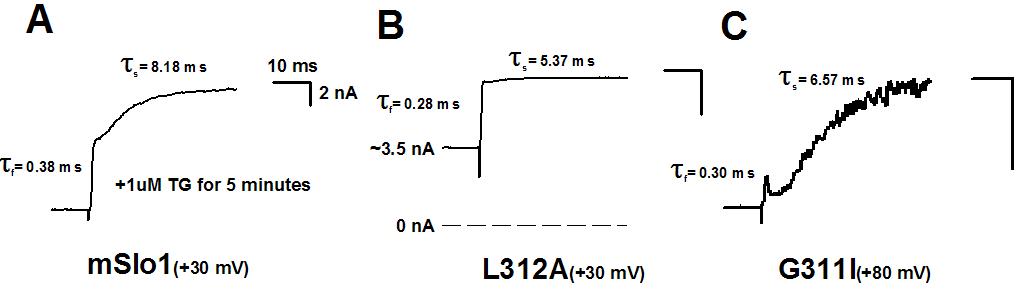


**sFig. 2**

**
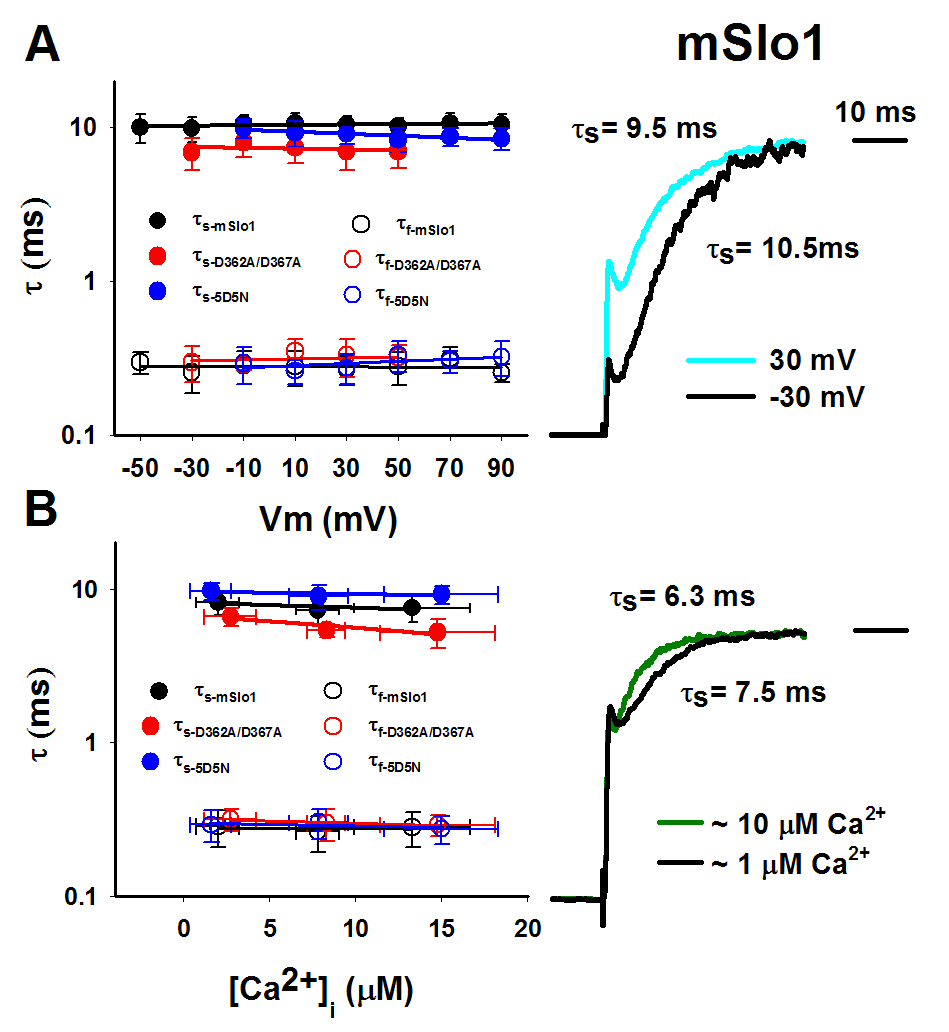
**

**sFig. 3**


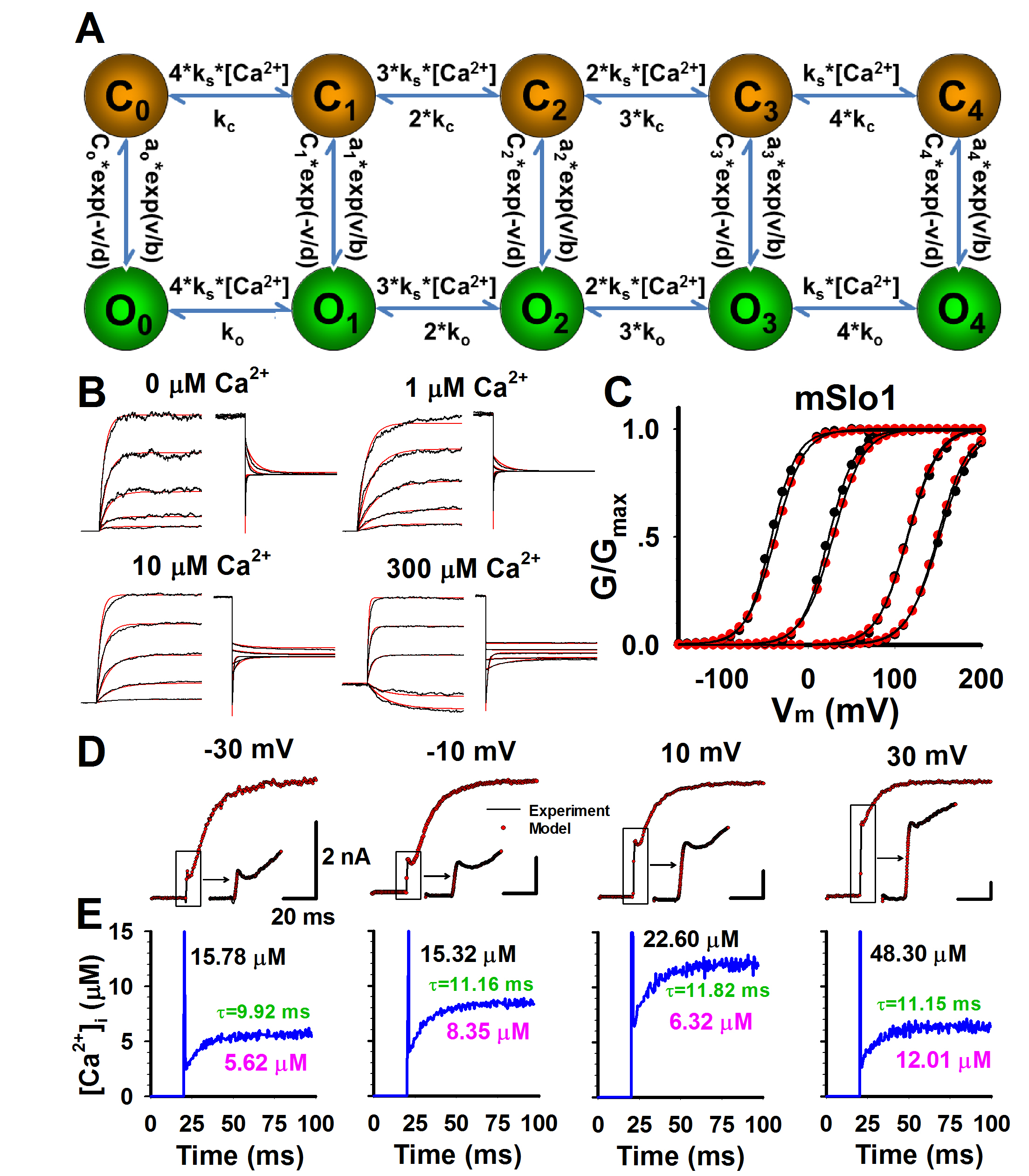


**sFig. 4**

**
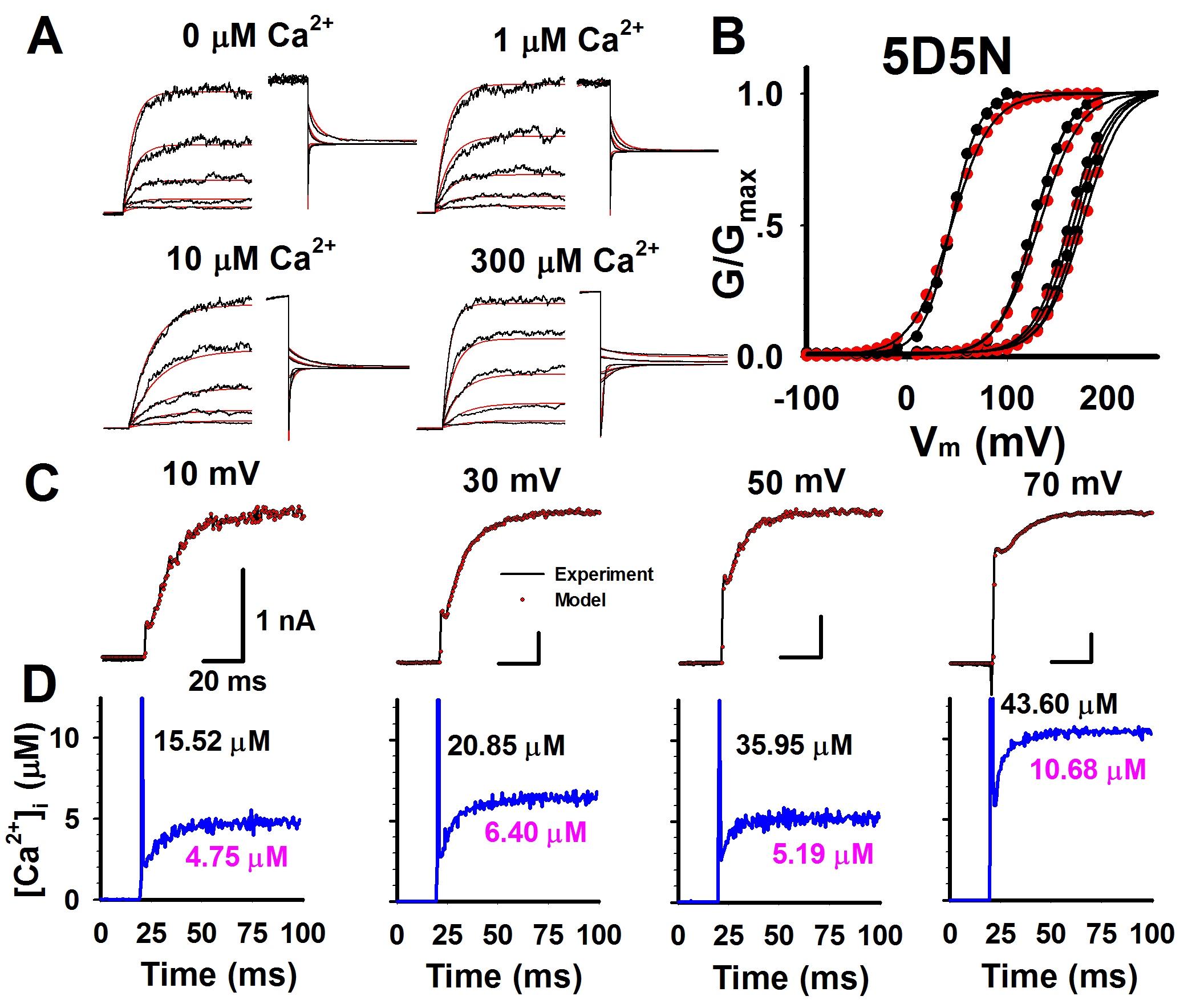
**

**sFig. 5**

**
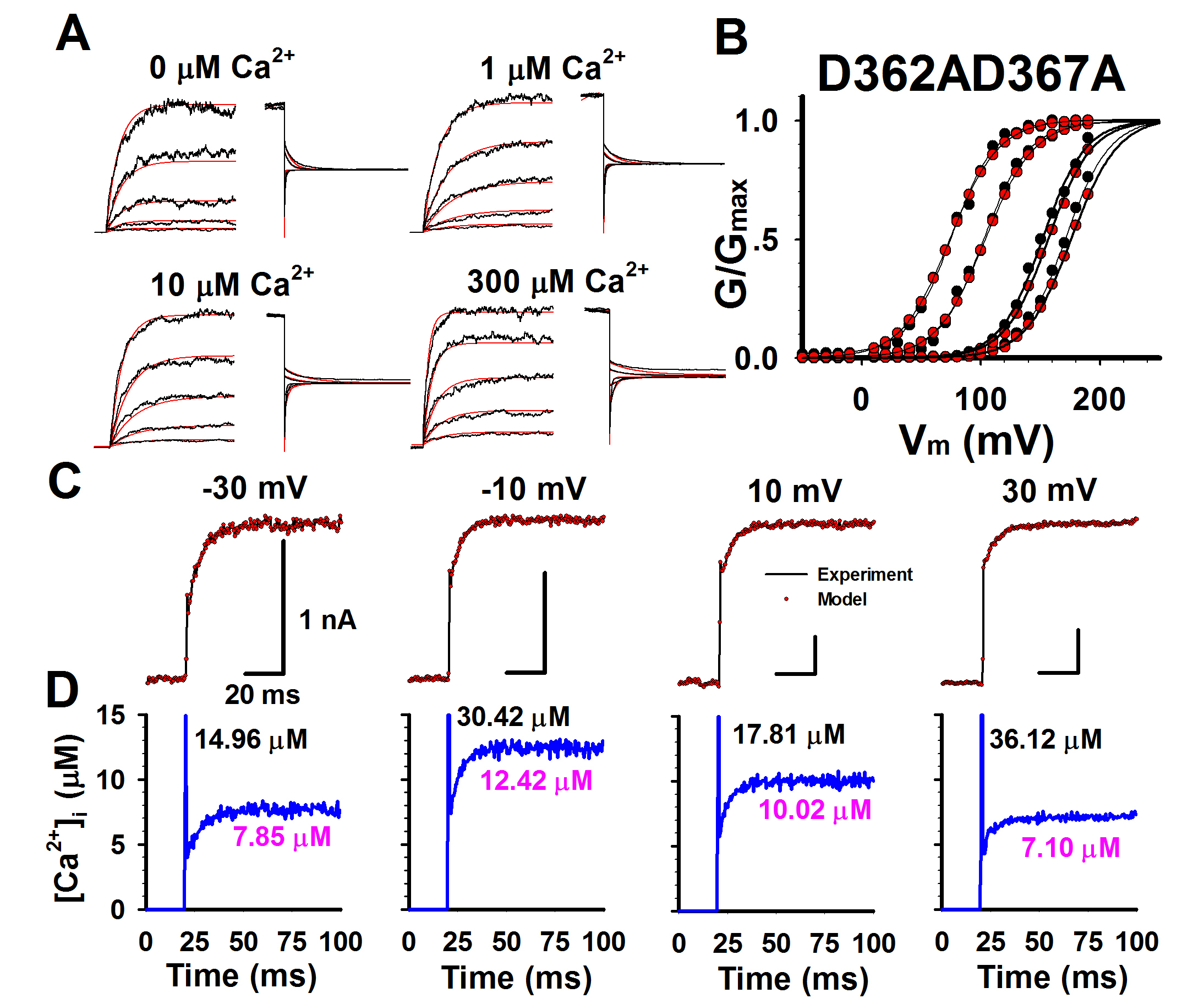
**

**sFig. 6**

**
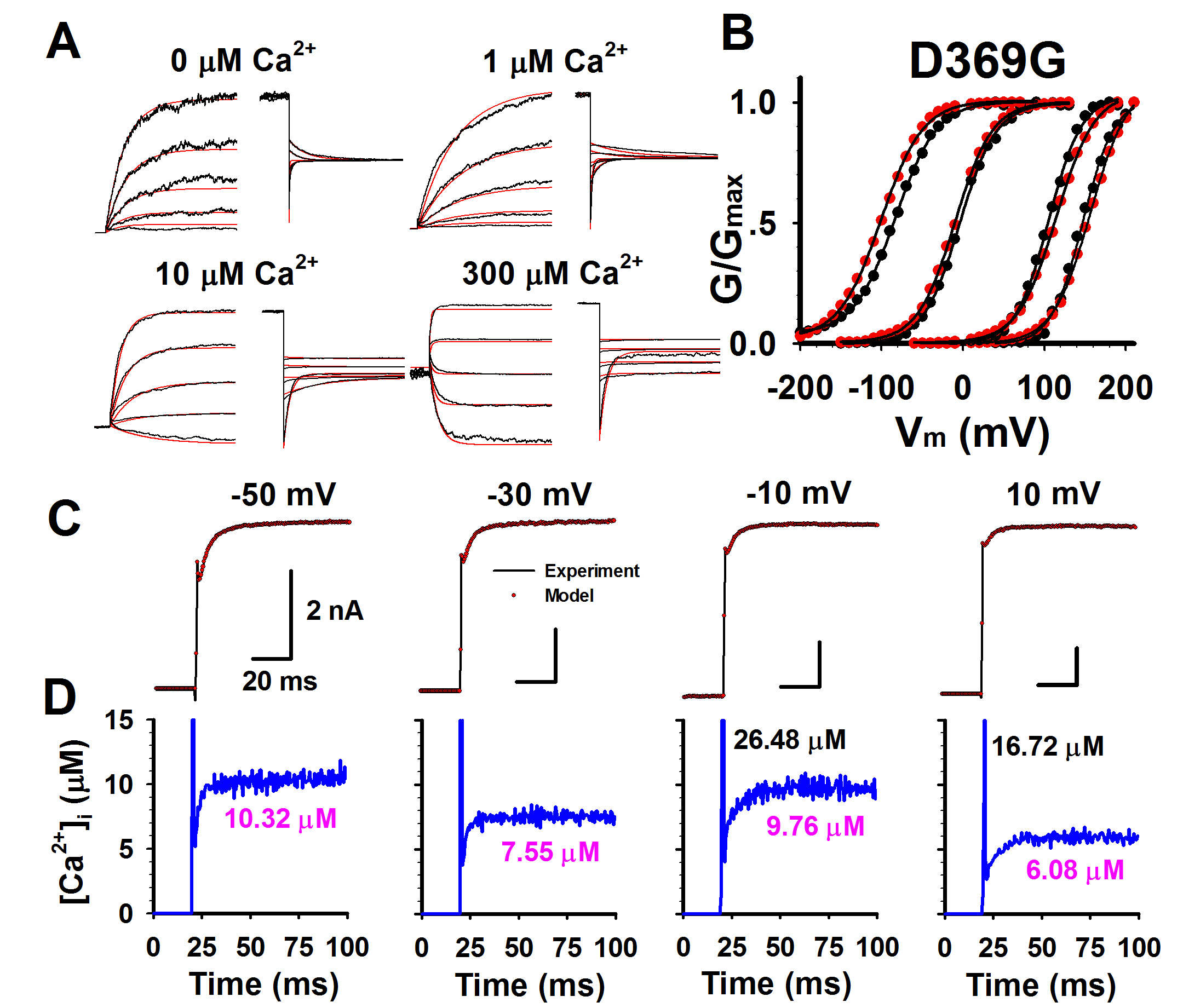
**

**sFig. 7**

**
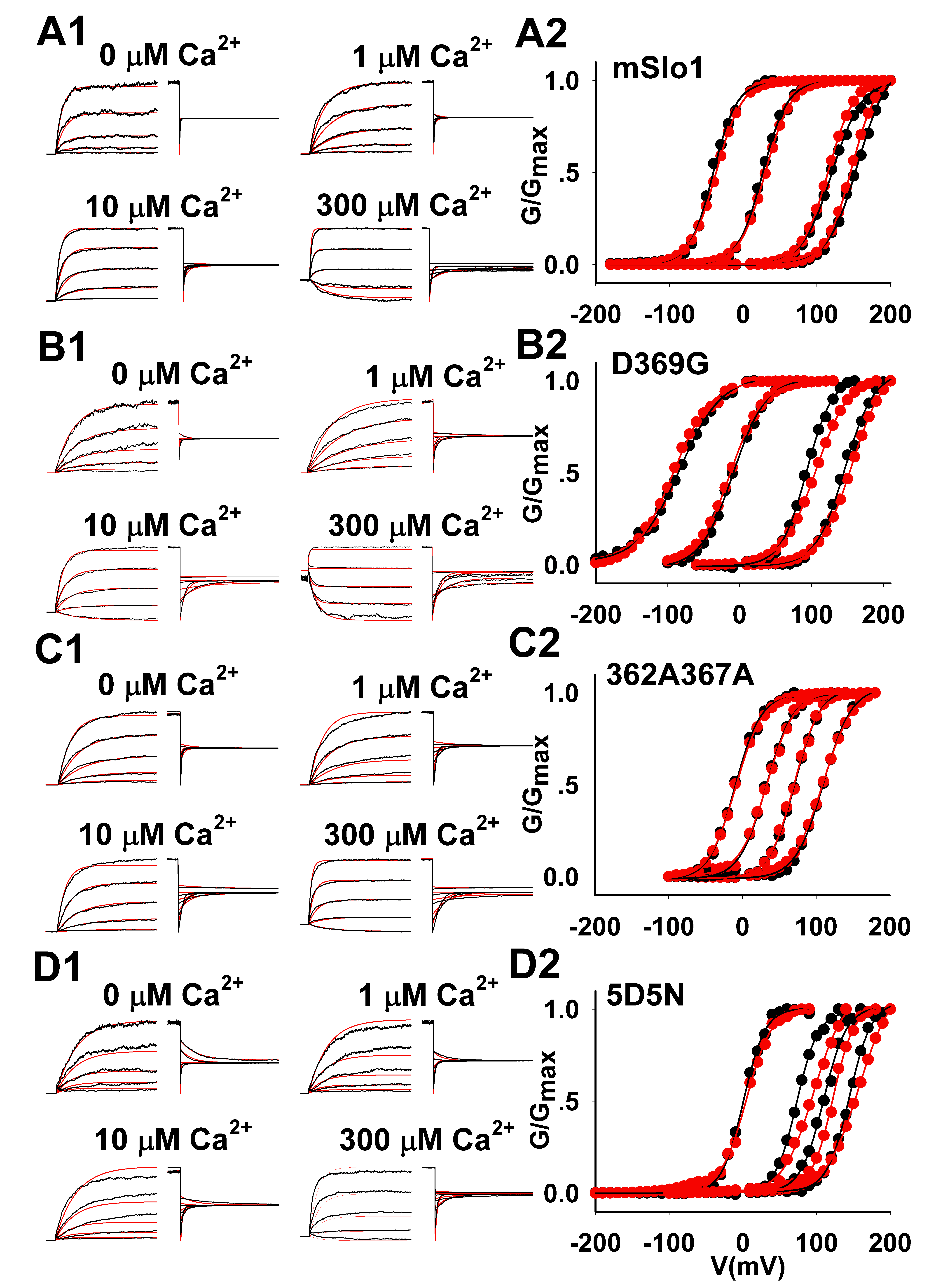
**

**sFig. 8**

**
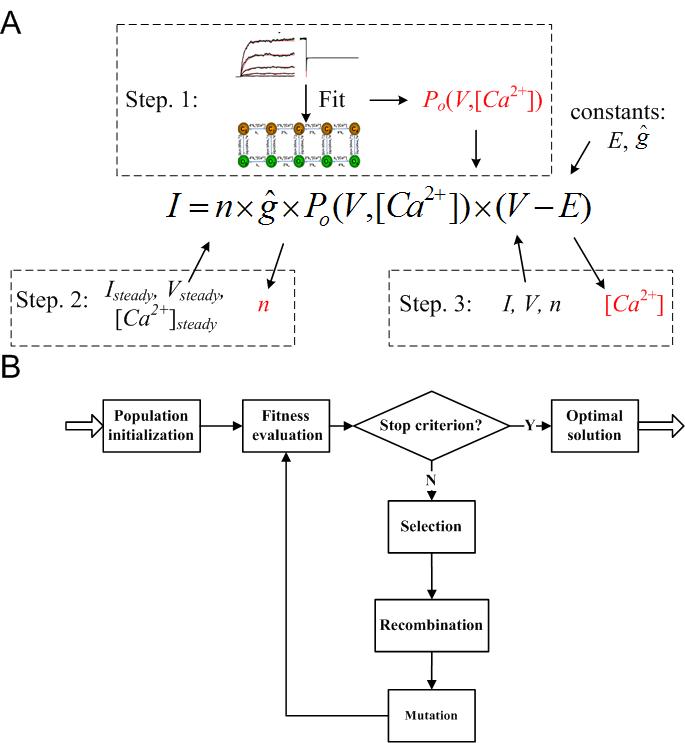
**

**sFig. 9**

**
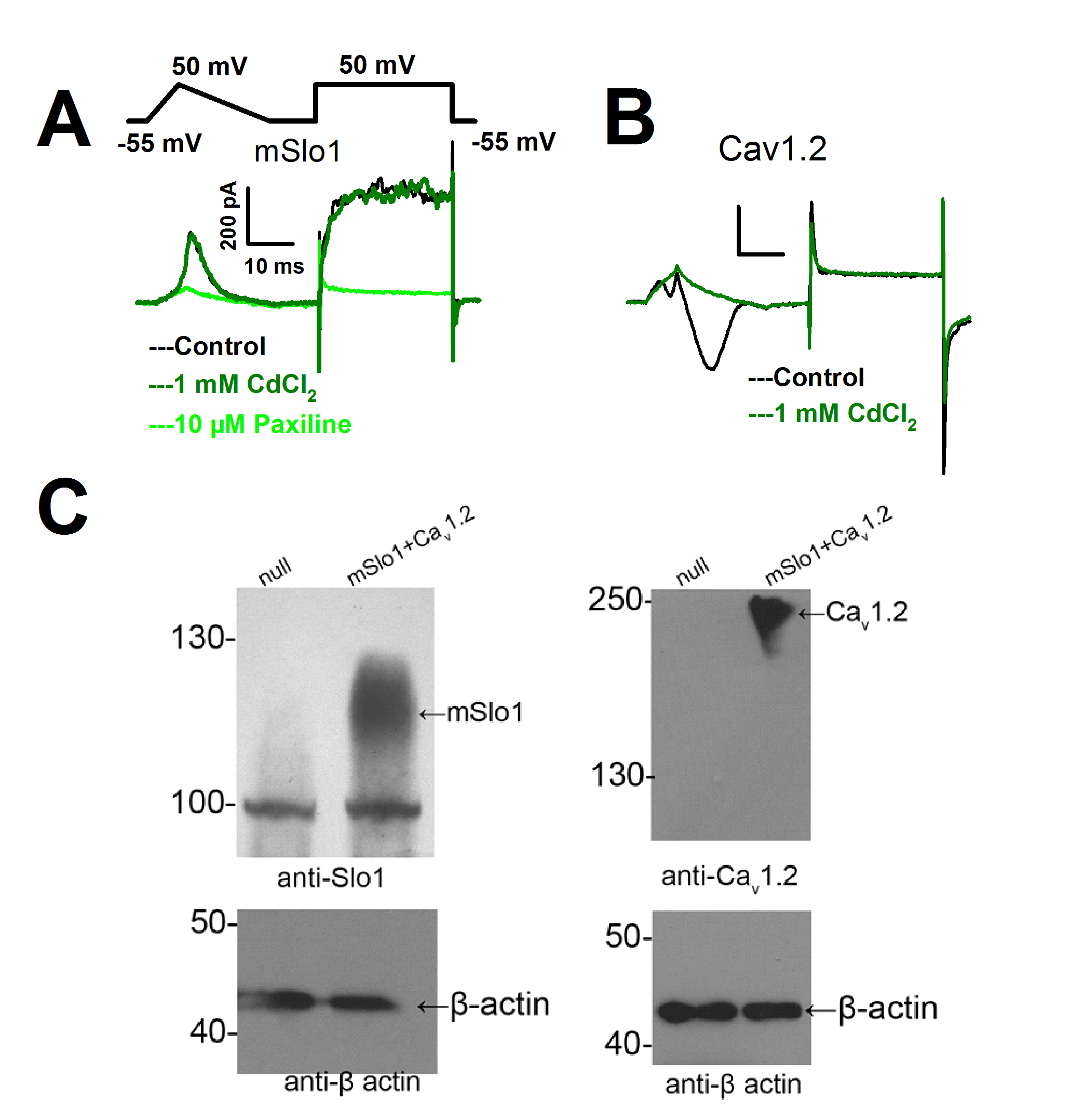
**

**Tables**

**sTable 1**

Apparent parameters in BK-type models

|  | mSlo1 | 5D5N | 362A/367A | D369G |
| --- | --- | --- | --- | --- |
| k_b_ | 1 | 1 | 1 | 1 |
| k_c_(μM) | 14.85 | 69.54 | 44.81 | 25.61 |
| k_o_(μM) | 1.77 | 11.27 | 12.06 | 1.66 |
| b(mV) | 32.29 | 34.44 | 33.60 | 42.79 |
| d(mV) | 70.79 | 72.92 | 71.06 | 60.73 |
| c_4_(ms^-1^) | 0.27 | 0.39 | 2.18 | 0.04 |
| c_3_(ms^-1^) | 0.65 | 0.07 | 0.06 | 0.16 |
| c_2_(ms^-1^) | 1.39 | 1.76 | 0.14 | 0.21 |
| c_1_(ms^-1^) | 0.95 | 0.08 | 1.93 | 0.07 |
| c_0_(ms^-1^) | 1.40 | 3.90 | 2.86 | 1.62 |
| a_4_(ms^-1^) | 1.12 | 0.11 | 0.11 | 3.42 |
| a_3_(ms^-1^) | 0.32 | 0.0028 | 0.0009 | 1.04 |
| a_2_(ms^-1^) | 0.08 | 0.0115 | 0.0001 | 0.08 |
| a_1_(ms^-1^) | 0.0066 | 0.0001 | 0.0019 | 0.0019 |
| a_0_(ms^-1^) | 0.0011 | 0.0006 | 0.0008 | 0.0028 |

**sTable2**

**k_b_ values of BK data at 30 mV. (*μM*^-1^*ms*^-1^).**

| No. | mSlo1 | 5D5N | 362A367A | D369G |
| --- | --- | --- | --- | --- |
| 1. | 0.1407 | 0.0567 | 0.1463 | 0.2506 |
| 2. | 0.1494 | 0.0523 | —— | 0.2666 |
| 3. | 0.1439 | 0.0561 | 0.1433 | 0.2429 |
| 4. | 0.2209 | 0.0594 | 0.1260 | —— |
| 5. | 0.2052 | —— | 0.1317 | —— |
| 6. | 0.2227 | 0.0596 | —— | 0.2789 |
| mean | 0.1805 | 0.0568 | 0.1368 | 0.2597 |
| std | 0.0398 | 0.0029 | 0.0096 | 0.0162 |

**sTable 3**

Physical parameters of BK-type models.

|  | mSlo1 | 5D5N | 362A/367A | D369G |
| --- | --- | --- | --- | --- |
| k_b_ | 0.1805 | 0.0568 | 0.1368 | 0.2597 |
| k_c_(μM) | 2.68 | 3.95 | 6.13 | 6.65 |
| k_o_(μM) | 0.32 | 0.61 | 1.65 | 0.43 |
| b(mV) | 32.29 | 34.44 | 33.60 | 42.79 |
| d(mV) | 70.79 | 72.92 | 71.06 | 60.73 |
| c_4_(ms^-1^) | 0.27 | 0.39 | 2.18 | 0.04 |
| c_3_(ms^-1^) | 0.65 | 0.07 | 0.06 | 0.16 |
| c_2_(ms^-1^) | 1.39 | 1.76 | 0.14 | 0.21 |
| c_1_(ms^-1^) | 0.95 | 0.08 | 1.93 | 0.07 |
| c_0_(ms^-1^) | 1.40 | 3.90 | 2.86 | 1.62 |
| a_4_(ms^-1^) | 1.12 | 0.11 | 0.11 | 3.42 |
| a_3_(ms^-1^) | 0.32 | 0.0028 | 0.0009 | 1.04 |
| a_2_(ms^-1^) | 0.08 | 0.0115 | 0.0001 | 0.08 |
| a_1_(ms^-1^) | 0.0066 | 0.0001 | 0.0019 | 0.0019 |
| a_0_(ms^-1^) | 0.0011 | 0.0006 | 0.0008 | 0.0028 |
